# Prediction and Validation of Gene-Disease Associations Using Methods Inspired by Social Network Analyses

**DOI:** 10.1371/journal.pone.0058977

**Published:** 2013-05-01

**Authors:** U. Martin Singh-Blom, Nagarajan Natarajan, Ambuj Tewari, John O. Woods, Inderjit S. Dhillon, Edward M. Marcotte

**Affiliations:** 1 Center for Systems and Synthetic Biology, Institute for Cellular and Molecular Biology, University of Texas, Austin, Texas, United States of America; 2 Department of Medicine, Karolinska Institutet, Solna, Stockholm, Sweden; 3 Department of Computer Science. University of Texas, Austin, Texas, United States of America; 4 Department of Statistics. University of Michigan, Ann Arbor, Michigan, United States of America; 5 Department of Chemistry and Biochemistry. University of Texas, Austin, Texas, United States of America; Institute for Research in Biomedicine, Spain

## Abstract

Correctly identifying associations of genes with diseases has long been a goal in biology. With the emergence of large-scale gene-phenotype association datasets in biology, we can leverage statistical and machine learning methods to help us achieve this goal. In this paper, we present two methods for predicting gene-disease associations based on functional gene associations and gene-phenotype associations in model organisms. The first method, the Katz measure, is motivated from its success in social network link prediction, and is very closely related to some of the recent methods proposed for gene-disease association inference. The second method, called Catapult (Combining dATa Across species using Positive-Unlabeled Learning Techniques), is a supervised machine learning method that uses a *biased* support vector machine where the features are derived from walks in a *heterogeneous* gene-trait network. We study the performance of the proposed methods and related state-of-the-art methods using two different evaluation strategies, on two distinct data sets, namely OMIM phenotypes and drug-target interactions. Finally, by measuring the performance of the methods using two different evaluation strategies, we show that even though both methods perform very well, the Katz measure is better at identifying associations between traits and poorly studied genes, whereas Catapult is better suited to correctly identifying gene-trait associations overall.

The authors want to thank Jon Laurent and Kris McGary for some of the data used, and Li and Patra for making their code available. Most of Ambuj Tewari's contribution to this work happened while he was a postdoctoral fellow at the University of Texas at Austin.

## Introduction

Correctly predicting new gene-disease associations has long been an important goal in computational biology. One very successful strategy has been the so-called guilt-by-association (GBA) approach, in which new candidate genes are found through their association with genes already known to be involved in the condition studied. This association can in practice be derived from many different types of data. Goh et al.[1] construct a network where genes are connected if they are associated with the same disease, whereas Tian et al.[2] combine protein interactions, genetic interactions, and gene expression correlation, and Ulitsky and Shamir[3] combine interactions from published networks and yeast two-hybrid experiments.

One of the most commonly used kinds of association is derived from direct protein-protein interactions, such as the ones curated by the Human Reference Protein Database (HPRD) [4]. The last few years have seen a number of methods, such as CIPHER [5], GeneWalker [6], Prince [7] and RWRH [8], that have extended the association from just direct protein interactions to more distant connections in various ways. One kind of network that has proven to be particularly useful for predicting biological function is the functional interaction network, where a pair of genes is connected based on the integrated evidence from a wide array of information sources, as seen by Lee at al.[9]. These have been used to associate genes with phenotypes in model organisms [10], [11] and in humans [12], [13]. A recently published network, HumanNet, has been used to refine predictions from genome-wide association studies [14]. Since functional gene interaction networks aggregate many different types of information, they can achieve much greater coverage than pure protein-protein interaction networks.

Alternatively, we can think of the gene-disease association problem as a *supervised learning problem*, where each gene-disease pair is represented by a number of derived features (explicitly or implicitly using a kernel function) and then a classifier is learned to distinguish “positive” (or *known*) associations from “negative” ones, using previously studied gene-disease associations, and *unknown* gene-disease pairs as training data. Such an approach is taken by the recent ProDiGe method [15], which integrates a wide variety of heterogeneous data sets and uses support vector machines (SVMs) to identify potential gene-disease associations.

In the past decades, the growth of gene-phenotype associations in model species has been explosive, which suggests an alternative way to find candidate genes for human diseases. McGary et al.[16] used this treasure trove of information to find surprising connections between model species phenotypes and human diseases by looking for pairs of human diseases and model phenotypes that share a higher than expected number of orthologous genes. In this way, a number of new, and often surprising, model systems were found for human diseases. For instance, the human neural crest related developmental disorder Waardenburg syndrome shares gene modules with gravitropism (the ability to detect up and down) in plants, and mammalian angiogenesis has been found to involve the same pathways as lovastatin sensitivity in yeast. This model species information represents yet another form of functional connection that can be used for gene-phenotype association.

In this paper, we first propose two distinct but related GBA methods. One is based on the Katz method [17] that has been successfully applied for link prediction in social networks. The method is based on integrating functional gene interaction networks with model species phenotype data and computing a measure of similarity based on walks of different lengths between gene and phenotype node pairs. The second method, which we call Catapult (Combining dATa Across species using Positive-Unlabeled Learning Techniques) is a supervised learning method, wherein we represent gene-phenotype pairs in a feature space derived from *hybrid* walks through the heterogeneous network used by Katz. The supervised learning method falls under a class of learning methods called *Positive-Unlabeled* learning methods (ProDiGe [15] also belongs in this class) since the learning task has only positive and *unlabeled* examples (and *no* negative examples). The method naturally generalizes the computation of Katz on a heterogeneous network by learning appropriate feature weights.

To determine if a computational method truly associates genes with diseases, biological validation of the predicted associations – often by knockout studies in model systems, or through sequencing of patients – is needed. Since these can be expensive and hard to do in a high throughput way, it is common to measure the performance of GBA methods through cross-validation. Recent work has shown that a large fraction of the performance of GBA methods can be attributed to the multifunctionality of genes [18]. A priori, it is not clear exactly how the construction of the training and the test data sets affects the measured performance of a method. We show that Katz and Catapult outperform the state-of-the-art, as measured by standard cross-validation. Furthermore, we show that standard cross-validation is not always an appropriate yardstick for comparing the performance of methods, and that when an alternative method for cross-validation is used — measuring how well the methods do in predicting genes that have no previous disease (or drug) associations, simpler walk-based methods often achieve better performance than supervised learning counterparts. We also observe that the qualitative performance of the methods correlates better with the latter evaluation strategy. We evaluate the two proposed methods, and compare to state-of-the-art network-based gene-disease prediction approaches on two completely distinct sources of data, namely OMIM phenotypes and gene-drug interactions.

## Results and Discussion

**Figure 1:**
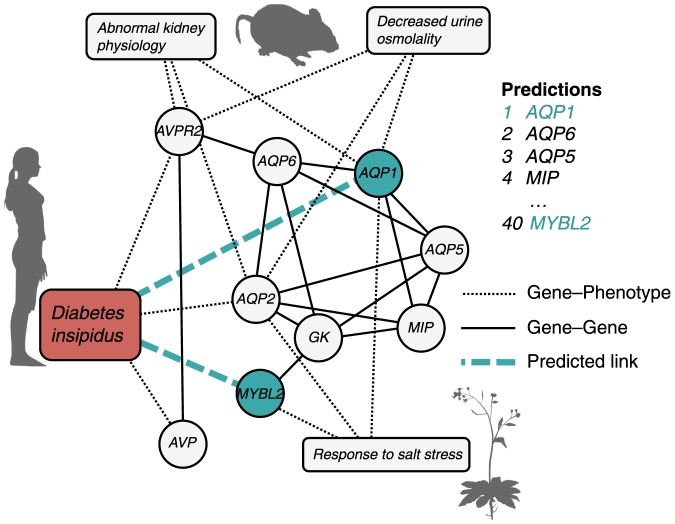
The combined network in the neighborhood of a human disease. The local network around the human disease diabetes insipidus and two genes highly ranked by Catapult, *AQP1* (top ranked candidate) and *MYBL2* (ranked as number 40). *AQP1* is ranked higher than *MYBL2* because there are more paths from diabetes insipidus to *AQP1* than to *MYBL2*, both through model organism phenotypes and through the gene--gene network. Only genes and phenotypes that are associated to both diabetes insipidus and the predicted genes *AQP1* and *MYBL2* are shown.

Conceptually, gene-disease association data can be thought of as a bipartite graph, where each gene and each disease is a node, and there is an edge between a gene node and a disease node if there is a known association between the gene and the disease. Similarly, we can form bipartite graphs from gene-phenotype association data of different species. By connecting a phenotype with a human gene if any *ortholog* of the human gene is associated with the phenotype, we obtain a bipartite network between human genes and phenotypes of different species. We can also obtain a phenotype-phenotype network for a given species, where a (weighted) edge 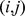 indicates that phenotype 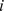 is “similar” to phenotype 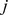. Adding a gene-gene interaction network completes a *heterogeneous* network of human genes and phenotypes in a wide variety of species. It is straightforward to define analogous heterogeneous network for gene-drug interactions, by replacing gene-disease associations data of humans with gene-drug associations. More limited heterogeneous networks have been considered previously in the context of gene-disease predictions, like the network of protein-protein interactions and human diseases [8], and in the context of gene-drug predictions [19]. Through a holistic view of the networks, otherwise unobserved ways of interactions between genes are revealed (via shared phenotypes), and independent information hidden in the model organism data can be leveraged for discovering novel associations between genes and human diseases or drugs. By integrating functional information from orthologs in multiple species, we also implicitly encode the functional relationships between homologous genes in the humans, which also contributes to our predictive performance. A visualization of the heterogeneous network consisting of gene-gene network and gene-phenotype networks of a few model species is presented in Figure 1.

In this setting, it is natural to view the problem of predicting gene-phenotype associations as a problem of finding similarities between nodes in a heterogeneous graph. Posing the problem in this way comes with the significant advantage that we can leverage a large body of work in machine learning and network analysis that deals with the problem of finding similar nodes in a graph [20], [21]. In particular, we adapt the Katz method [20] to the heterogeneous setting. As an extension of this work, we also introduce a supervised learning framework, Catapult. Catapult learns the importance of features associated with node pairs, where the features are derived from walk-based similarity measures between nodes.

### Katz on the heterogeneous network

The Katz measure is a graph-based method for finding nodes similar to a given node in a network[17]. It has been shown to be successful for predicting friends in social networks [20]. In this paper, we show the effectiveness of the method for the task of recommending genes for a given phenotype or drug. Suppose we are given an undirected, unweighted graph with a (symmetric) adjacency matrix 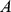, where 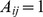 if node 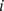 and node 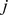 are connected, and 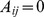 otherwise. One way to find the similarity between two (not necessarily connected) nodes 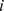 and 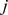 is to count the number of *walks* of different lengths that connect 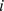 and 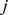. This has a natural connection to matrix powers since 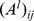 is exactly the number of walks of length 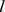 that connect 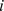 to 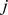. Hence 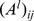 gives a measure of similarity between the nodes 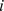 and 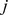. We want to obtain a single similarity measure that summarizes the similarities suggested by different walk lengths. For example, we could choose any sequence 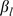 of non-negative coefficients and define the similarity
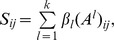
where 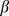 is a constant that dampens contributions from longer walks. In matrix notation, the similarity matrix 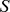 (that captures similarities between all pairs 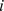 and 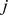) may be written as:
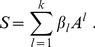
(1)As observed by a recent survey article [21], we can regard 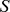 as a matrix function 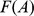 where 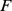 is defined through the series expansion in (1). Note that we may allow 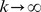, as long as 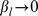 as 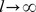. Specific choices for 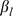 yield a variety of concrete similarity measures. A choice of 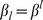 leads to the well-known Katz measure [17]:
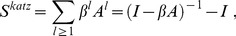
(2)where 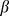 is chosen such that 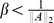. In the case where the connections in the graph are weighted such that 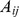 is the strength of the connection between nodes 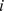 and 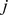, we can generalize the idea of walks using this matrix framework, by simply using the weighted adjacency matrix instead of the binary matrix. Different ways of constructing the matrix 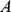 together with the appropriate normalizations of the matrix lead to methods of the type used by PRINCE [7], RWRH [8], GeneMANIA [11], and by the famous PageRank algorithm used for web page ranking [22]. However, we do not necessarily have to consider sum over infinitely many path lengths. Paths of shorter lengths often convey more information about similarity between a given pair of nodes, and contributions from longer paths become insignificant. This suggests that we can consider a finite sum over path lengths, and typically small values of 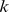 (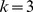 or 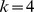) are known to yield competitive performance in the task of recommending similar nodes [23].

Let 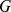 denote the gene-gene network, let 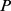 denote the bipartite network between genes and phenotypes, and let 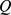 denote the phenotype-phenotype network. In particular, 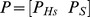 is a composite of the gene-disease network of humans, written 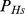, and the gene-phenotype networks of other species, written 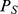. Similarly,
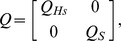
where 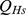 is the similarity matrix of human diseases, and 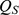 is that of phenotypes of other species. In our experiments, we set 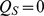, since we do not have information about similarity between phenotypes of other (non-human) species. The construction of the matrices 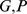 and 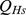 will be discussed in detail in the Methods section. We form a *heterogeneous* network over the gene and phenotype nodes, similar to RWRH (which we will review briefly in the Methods section). The adjacency matrix of the heterogeneous network may be written as:
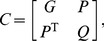
(3)Recall the general formula for the truncated Katz similarity measure when specialized to the combined matrix 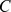:
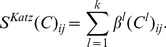
(4)From here on, we will drop the “truncated” and refer to this simply as the Katz measure. Note that for smaller values of 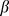, higher order paths contribute much less. It has been shown that restricting the sum to a small 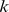, *i.e.* a few higher order paths, works well in practice, in network link prediction and recommender systems[23]. Letting 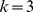, the block of the Katz score matrix 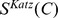 corresponding to similarities between gene nodes and human disease nodes, written 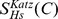, can be expressed as:
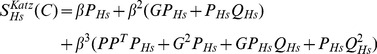
(5)where 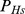 and 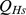 denote the gene-phenotype and phenotype-phenotype networks of humans respectively. We use equation (5) to compute scores for the Katz method in our experiments.

In case of the drugs data set, we use the gene-drug network 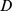, instead of 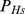 in equation (5). We do not have similarity information for drugs, and so we set 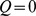 for experiments on the drug data set. Nonetheless, we use phenotype information from multiple species, in the composite matrix 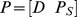, in order to infer similarities between gene and drug nodes, where the matrix 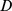 represents the known associations between genes and drugs (replacing 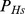 above in the gene-disease example).

### CATAPULT: A supervised approach to predicting associations

The fixed choice of parameters involved in the Katz and random walk based approaches, as in equation (4), provides a reasonable initial approach. However, to improve performance we would like to *learn* the weights based on the heterogeneous network itself. That is, instead of using the exponential damping weights 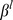, we can try to learn the relative importance of paths of different lengths. To this end, we frame the problem of predicting potential gene-phenotype associations as a supervised learning problem, in which we want to learn a classifier (or ranking) function whose input space consists of gene-phenotype pairs and output is a score for each gene-phenotype pair. In particular, by appropriately defining the feature space for gene-phenotype pairs, we will see that learning a classifier in the constructed feature space is tantamount to learning coefficients for Katz on the heterogeneous network computed as in equation (5).

For any given phenotype, it is very hard to verify that a gene is *not* associated in some way with the phenotype. Our learning strategy is therefore guided by the fact that *absence of evidence is not evidence of absence*. While a biological experiment can give clear evidence for the existence of a certain gene–phenotype association, a lack of evidence for a connection does not imply that such a connection does not exist. Biologists therefore tend to report positive associations between genes and phenotypes. However, the reported list of gene–phenotype associations is not exhaustive. Because negative associations rarely get reported, we treat all gene–phenotype pairs for which no positive association has been reported as unlabeled, with the prior assumption that most of them are in fact negative associations. Our data set therefore has the following two key characteristics:

1. For each phenotype, we only have a partial list of the associated genes. That is, we only know of positive associations; we do not have negative associations available to us.
2. There is a large number of unlabeled gene-phenotype pairs with the prior knowledge that most of them are, in fact, negative associations.

Classical supervised learning methods require both positive and negative examples, and therefore fall short in our case. Positive-Unlabeled learning (PU learning for short) methods are natural for this setting. The general idea of PU learning methods is to identify a set of negatives from the unlabeled examples and train a supervised classifier using the positives and the identified negatives. Liu et al.[24] study different ways of choosing negatives from unlabeled examples. Biologists believe that only a few of the large number of *unobserved* associations are likely to be positive. A random sample is likely to consist mostly of negatives, which suggests that we could randomly choose a set of examples and use the random sample as “negative” examples to train a supervised classifier. As the examples are *not* known to be negative, it may be helpful to allow the classifier to not heavily penalize the mistakes on “negatives” in the training phase. We therefore learn a *biased* support vector machine classifier using the positive associations and a *random* sample of unlabeled associations. Recently, Mordelet et al.[25] proposed ProDiGe that also uses a random sample of unlabeled examples as a negative sample to train a biased support vector machine against a set of known positives. The support vector machine is biased in the sense that false negatives (known positives classified as negatives) are penalized more heavily than the false positives (“negatives” classified as positives). The bias makes sense because the positive examples are known to be positive, while the negatives are arbitrary and hence false positives are not to be penalized too heavily. Note that, in principle, we could use any PU learning method (for instance, the weighted logistic regression model proposed in [26]) to obtain a classifier for gene-phenotype pairs.

**Figure 2:**
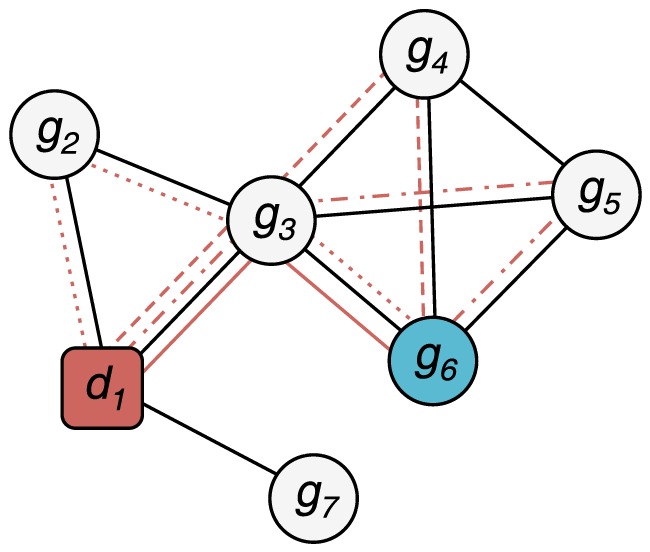
Katz features are derived by constructing walks of different kinds on the graph. In the figure above, the disease node 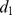 is connected to the gene node 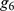 by one walk of length 2 (solid red line) and three walks of length 3 (dotted, dashed and dashdotted red lines). This can be quickly calculated from the adjacency matrix 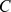 of the graph: If 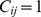 when there is a link between nodes 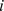 and 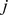, and 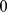 otherwise, the number of paths of length 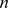 between genes 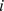 and 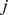 is 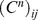. In the example above, 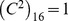 and 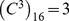.

Figure 2 demonstrates simple walk-based features derived from the heterogeneous network. Gene-phenotype pairs are represented using the walk-based features, and classified using a biased support vector machine in our Catapult algorithm.

### Functional data outperforms protein-protein interactions

To see how the Katz and Catapult methods compare to the state-of-the-art, we measured their recovery of genes using a cross-validation strategy similar to the one used by Mordelet and Vert [15], on two different data sets — gene-disease associations from the Online Mendelian Inheritance in Man (OMIM, [27]), and a recent drug-gene interaction data set [19]. These data sets can both be thought of as large collections of gene-trait pairs, either as gene-disease pairs for the OMIM data, or target-drug pairs for the drug data set.

We compared Katz and Catapult to four recent methods:

1. The recently proposed **ProDiGe** method [15], which is a support vector machine based method that calculates similarity scores for gene pairs using a wide variety of information sources including 21 different gene-gene functional interaction networks and phenotype similarities.
2. **RWRH** [8], which, like Katz uses walks on a heterogeneous gene-disease graph to prioritize genes. It differs from the Katz method chiefly in how the heterogeneous network is normalized. We discuss the relationship in more detail in Text S1.
3. We include **PRINCE** [7] for completeness, since it is the state-of-the-art to which both RWRH and ProDiGe were compared.
4. Finally, some recent work [18] has shown that simply by ranking based on the degree centrality of a gene (how often it interacts with other genes, or is involved in diseases) can be a very competitive ranking strategy. We therefore predict genes for diseases (or drugs) using a simple degree-based list, where all genes are ranked by how many diseases (drugs) they are known to be connected to, *regardless* of which disease (drug) the predictions are made for.

For cross-validation, we use the same testing framework as the one used by Mordelet and Vert [15]: split the known gene-trait pairs into three equally sized groups. We hide the associations in one group and run our methods on the remaining associations, repeating three times to ensure that each group is hidden exactly once. A clarification on the correctness of the cross-validation procedure while using heterogeneous information sources is in order: We do not create any gene-phenotype associations beyond those that are directly experimentally observed. We incorporate data from other species by orthology, but link it directly to the corresponding human gene, and do not create gene nodes for the orthologous species. Moreover, the only phenotype-phenotype linkages we include are across human disease pairs and are derived solely from MimMiner text mining of OMIM records [28] —there is no incorporation of gene sequence information whatsoever, only a measure of similarity of the clinical features of the diseases. Thus, there is no circularity created by homology in the data during cross-validation. For each trait in our data set, we order all the genes by how strongly the method predicts them to be associated with the trait. Finally, for every gene-trait pair 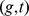 in the hidden group we record the rank of the gene 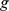 in the list associated with trait 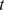. We use the cumulative distribution of the ranks as a measure for comparing the performances of different methods, i.e. the probability that the rank (at which hidden gene-trait pair is retrieved) is less than a threshold 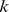. The motivation of using this performance measure is that a method that has a higher probability of recovering a true association in the top-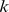 predictions for a given disease is desired. Recent methods including ProDiGe[15] have adopted this performance measure for comparison.

**Figure 3:**
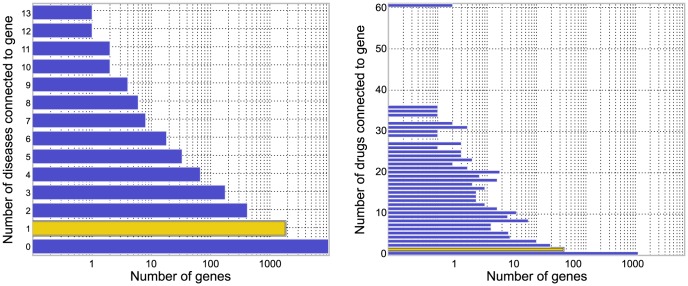
Empirical cumulative distribution function for the rank of the withheld gene under cross-validation. Left panel corresponds to evaluation of OMIM phenotypes, and the right corresponds to drug data. The vertical axis shows the probability that a true gene association is retrieved in the top-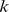 predictions for a disease. Katz and Catapult methods use all species information, and the **HumanNet** gene network. PRINCE and RWRH methods are implemented as proposed in [7] and [8] respectively, using the **HPRD** gene network. ProDiGe method is implemented as discussed in Methods section. Catapult (solid red) does much better across the data sets under this evaluation scheme. In general, the methods get high precision rates in case of the drug data. PRINCE method that does not allow walks through species phenotypes, and OMIM phenotypes in particular, performs much worse than other random-walk based methods. ProDiGe allows sharing of information between phenotypes using the similarities between OMIM phenotypes and performs reasonably well, whereas there is no such sharing possible in case of the drug data due to the absence of drug similarities. The simple degree-based method performs poorly in general. ProDiGe and PRINCE essentially use only the gene network information in case of the drug data.

The results are presented in Figure 3. Note that the vertical axis in the plots give the probability that a true gene association is recovered in the top-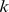 predictions for various 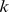 values in the horizontal axis. For example, we observe that the Katz method has over 15% probability of recovering a true gene in the top-100 predictions for a disease, whereas PRINCE is only 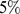 likely to retrieve a true association in the top-100 predictions. Under this evaluation method, both Katz and Catapult, which make use of much more extensive data sets than the other methods, are quite likely to recover the hidden gene among the top 100 genes. As can be seen from Figure 3, Katz and Catapult perform better than *any* of the previously studied state-of-the-art gene-disease association methods for the OMIM data set. Catapult also performs well on the drug data set, ranking the hidden gene 14th or lower a remarkable 50% of the time. RWRH, which like Katz and Catapult is a walk based method that allows paths through the gene-disease (or, for the drug data set, gene-drug) network, also does quite well.

ProDiGe allows sharing of information between phenotypes using the similarities between OMIM phenotypes, and also integrates a wide variety of functional information in a supervised machine learning framework and performs reasonably well on the OMIM phenotypes. The PRINCE method, which allows some sharing of information between OMIM diseases that are phenotypically similar, performs worse than the other random-walk based methods. Since we have no similarity information available for the drug data, ProDiGe and PRINCE essentially use only the gene similarity information in the drug data case. Notice that the simple degree-based method does the worst of all methods in case of OMIM phenotypes, which suggests that recommendations given by walk-based methods are more relevant and differ significantly from simple ranking by number of known associations.

**Figure 4:**
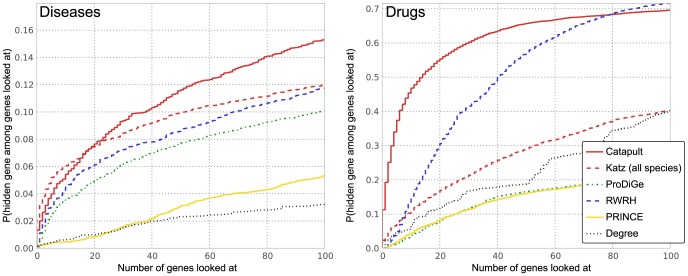
Comparison only using HumanNet. Empirical cumulative distribution function for the rank of the withheld gene under cross-validation. Left panel corresponds to evaluation of OMIM phenotypes, and the right corresponds to drug data. The vertical axis shows the probability that a true gene association is retrieved in the top-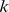 predictions for a disease. Katz and Catapult methods use all species information, and all the methods use the **HumanNet** gene network. PRINCE and RWRH methods are implemented as proposed in [7] and [8] respectively, but using **HumanNet**. ProDiGe method is implemented as discussed in Methods section. Again, as in Figure 3, Catapult (solid red) does the best. An important observation to be made from the plots is that PRINCE and RWRH methods perform relatively much better than in Figure 3, where HPRD network was used. (Note that there is no change to the ProDiGe, Katz and Catapult methods; they have identical settings as in Figure 3).

To see if the improvement in performance of Katz and Catapult stems from the more extensive network used, or, in Catapult's case, the increased sophistication of the machine learning method, we evaluated network based RWRH and PRINCE methods using the more extensive HumanNet network instead of the HPRD network originally used. As can be seen in Figure 4, Catapult still does better than the previous state-of-the-art using this cross-validation framework, consistently in both the OMIM and drug data sets.

#### Precision-Recall measure

**Figure 5:**
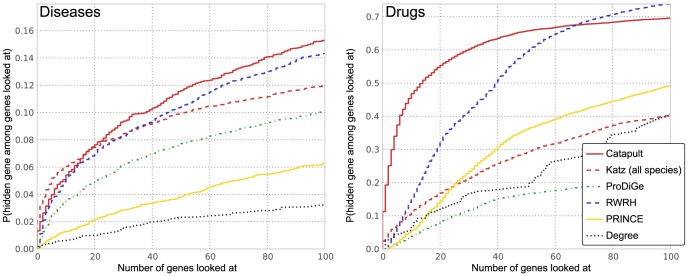
Precision-Recall curves for three-fold cross validation. Left panel corresponds to evaluation of OMIM phenotypes, and the right corresponds to drug data. The vertical axis shows the precision rate, i.e. fraction of true positives in the top-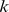 predictions. The horizontal axis shows the recall rate, i.e. ratio of true positives recovered in the top-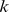 predictions to the total number of positives for a phenotype (or a drug) in the hidden set. The plots show precision-recall values at various thresholds 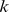, in the range 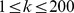 and the value at a given 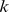 is averaged over all the phenotypes (drugs). The plots use the same experimental setup as in Figure 4, and we observe that the comparisons illustrated by precision-recall measure are consistent with the rank cdf measure in Figure 4.

**Figure 6:**
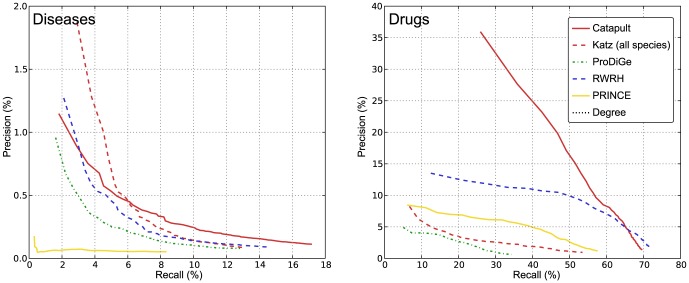
Empirical cumulative distribution function for the rank of withheld singleton genes. Left panel corresponds to evaluation of OMIM phenotypes, and the right corresponds to drug data. The vertical axis shows the probability that a true gene association is retrieved in the top-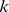 predictions for a disease. The Katz and Catapult methods use all species information, and all the methods use the **HumanNet** gene network. PRINCE and RWRH are implemented as proposed in [7] and [8] respectively, but using the **HumanNet** gene network. The ProDiGe method is implemented as discussed in the Methods section. We have not included the degree based list from Figure 4, since all the singleton genes are always given degree 0 during cross-validation. Catapult (solid red) does much better than ProDiGe (the only other supervised method) but does worse compared to walk-based methods than in Figure 4 (that uses the same setting for all the methods). PRINCE and ProDiGe are consistent with (and sometimes perform slightly better than) the full cross-validation evaluation. RWRH and the Katz measure perform better than the supervised learning methods ProDiGe and Catapult in this evaluation scheme. The fact that PRINCE performs so well on singletons in the drug data case is surprising, given that the only information it uses is the HumanNet gene network.

We also evaluate and compare the different methods using the more familiar precision and recall measures. Precision measures the fraction of true positives (genes) recovered in the top-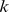 predictions for a trait. Recall is the ratio of true positives recovered in the top-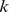 predictions for a trait to the total number of true positives in the hidden set. The plot of precision vs recall rates for different values of thresholds 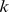 ranging 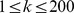 is presented in Figure 5. Note that we are more interested in small values of 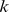, similar to the results corresponding to the rank cdf measure presented in Figures 3, 4 and 6. Our experimental setup is identical to that in Figure 4, i.e. using HumanNet for all the relevant methods. The comparisons on both OMIM and drug data sets observed from Figure 5 are identical to Figure 4. In particular, Catapult performs much better in case of the drug data, and is competitive to Katz and RWRH methods at very small values of 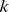 (in the range 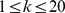) and performs much better outside the regime, in case of the OMIM data. Note that we observe identical behavior for Catapult in the left panel of Figure 4. The performance of the other methods are similarly consistent across the two performance measures.

#### Focusing on the gene linkage neighborhood

**Figure 7:**
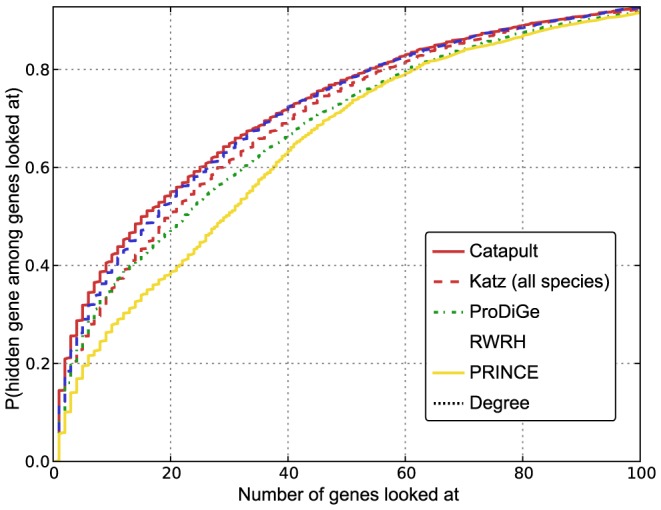
Empirical cumulative distribution function for the rank of withheld genes from OMIM phenotypes, restricted to genes in a small linkage neighborhood of the withheld genes. The vertical axis shows the probability that a true gene association is retrieved in the top-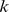 predictions for a disease. The Katz and Catapult methods use all species information, and all the methods use the **HumanNet** gene network. PRINCE and RWRH are implemented as proposed in [7] and [8] respectively but using the **HumanNet** gene network. The ProDiGe method is implemented as discussed in the Methods section. We observe that Catapult performs the best. RWRH and Katz methods are competitive as well. The results are consistent with our observations from Figure 4.

The results so far are on measuring how well a candidate gene can be predicted *genome wide*. Another common scenario is where a linkage interval is known for a disease, but the causal disease linked gene has not been identified. To simulate this setting, we use an approach similar to the one taken in [7]. For each known gene-disease pair 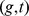, we construct a simulated linkage interval by taking all genes within 10 million basepairs from either end of the gene 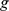 (containing a median of 84 genes), and record the rank at which gene 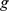 is predicted for trait 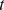 when 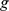 is masked. As can be seen in Figure 7, Catapult again performs the best.

#### When do Catapult and GBA methods fail?

**Figure 8:**
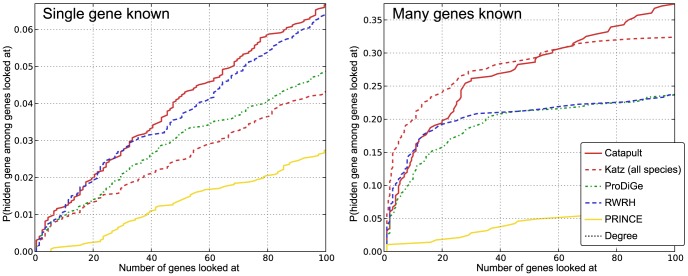
Empirical cumulative distribution function for the rank of withheld genes from OMIM phenotypes with single known gene (left panel) and more than one known gene (right panel). The vertical axis shows the probability that a true gene association is retrieved in the top-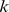 predictions for a disease. The Katz and Catapult methods use all species information. PRINCE and RWRH are implemented as proposed in [7] and [8] respectively, using **HPRD** network. The ProDiGe method is implemented as discussed in the Methods section. In case of phenotypes with only one known gene (left panel), the only information is the phenotype-phenotype similarity. From the left panel, we note that all network-based methods perform poorly. Nonetheless, we observe a gradation in the performances of different methods, and that Catapult does slightly better. All the methods do substantially better on phenotypes with more than one known gene (right panel).

It is important to know if there is a set of phenotypes for which Catapult, and other network-based GBA methods, do not perform well. To understand the same, we looked at the phenotypes for which Catapult attained the poorest (average) recall rate. In particular, we ordered the phenotypes by the (mean) recall rate for the hidden genes (in three-fold validation). We find that the bottom-most phenotypes in the ordering are precisely the ones for which there is *only one* known gene. Note that the training data for these phenotypes *did not* have the known gene. The only information for the phenotypes comes from the phenotype-phenotype similarities. However, there are some phenotypes for which even phenotype similarities are not known. In such cases, all GBA or network-based methods will fail. The results are presented in Figure 8 (left panel). We observe that all network-based methods perform poorly. Nonetheless, we observe a gradation in the performances of different methods, and Catapult does slightly better. The difference in performance is not surprising given that some methods use the heterogeneous network fully ( Catapult and Katz) but others only partially (ProDiGe, PRINCE and RWRH). All the methods do substantially better on phenotypes with more than one known gene (right panel). A qualitative analysis of the methods discussed next, however, shows that the boost in performance may not necessarily reflect that the predictions made by the methods are pertinent to the phenotypes. This connection between the size of sets and how easy they are to predict has also been observed in the context of GO annotations, see for instance [29].

### Top predictions by supervised methods are enriched for highly connected genes

**Figure 9:**
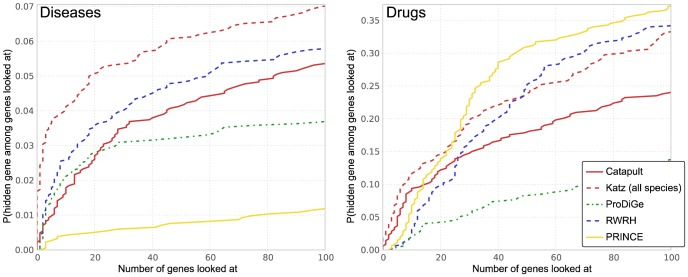
Distribution of the number of known genes in OMIM diseases (left) and drugs (right). The bar corresponding to the genes on which we did the singleton validation is shown in yellow.

To get a qualitative view of how the connectedness of genes influences the rankings, we plotted the degree distribution of the genes in the OMIM and the drug data sets in Figure 9, and compared the results with the list of top candidates from Catapult (see Table 1) and Katz (see Table 2).

**Table 1:** Top predictions for Catapult.

Leukemia	Alzheimer disease	Insulin resistance	Prostate cancer
MIM:601626	MIM:104300	MIM:125853	MIM:176807
***FGFR3*** (2261)	*ACE2* (59272)	***INSR*** (3643)	***TP53*** (7157)
***FGFR2*** (2263)	*COL1A1* (1277)	*INS* (3630)	***RB1*** (5925)
***KRAS*** (3845)	*COL1A2* (1278)	***PTEN*** (5728)	***CTNNB1*** (1499)
***TP53*** (7157)	***KRAS*** (3845)	***TP53*** (7157)	***BRCA1*** (672)
***EGFR*** (1956)	***EGFR*** (1956)	***CTNNB1*** (1499)	***KRAS*** (3845)
***FGFR1*** (2260)	***TP53*** (7157)	***KRAS*** (3845)	***PIK3CA*** (5290)
*PTPN11* (5781)	*AGT* (183)	***AKT1*** (207)	***AKT1*** (207)
***CTNNB1*** (1499)	*PLAT* (5327)	***CREBBP*** (1387)	***INSR*** (3643)
***INSR*** (3643)	*APOE* (348)	***EGFR*** (1956)	***NRAS*** (4893)
***CREBBP*** (1387)	*PTGS2* (5743)	***PIK3CA*** (5290)	*RAD51* (5888)

Schizophrenia	Breast cancer	Gastric cancer	Colorectal cancer
MIM:181500	MIM:114480	MIM:137215	MIM:114500
*BDNF* (627)	***PTEN*** (5728)	***FGFR3*** (2261)	***KRAS*** (3845)
*NRG1* (3084)	***RB1*** (5925)	***FGFR1*** (2260)	***PTEN*** (5728)
*CBS* (875)	***NRAS*** (4893)	***NRAS*** (4893)	***CTNNB1*** (1499)
*NOS2* (4843)	***BRCA1*** (672)	***HRAS*** (3265)	***HRAS*** (3265)
*MTR* (4548)	***HRAS*** (3265)	***EGFR*** (1956)	***CREBBP*** (1387)
*HTR2C* (3358)	***INSR*** (3643)	*ERBB3* (2065)	***RB1*** (5925)
*HTR2B* (3357)	***CTNNB1*** (1499)	***CTNNB1*** (1499)	***FGFR3*** (2261)
*SLC6A4* (6532)	***EGFR*** (1956)	*BRAF* (673)	***INSR*** (3643)
***FGFR2*** (2263)	***FGFR3*** (2261)	***PTEN*** (5728)	***EGFR*** (1956)
*MAT1A* (4143)	***FGFR2*** (2263)	***TP53*** (7157)	***FGFR2*** (2263)

Top 10 predictions not in the training set by Catapult for the eight OMIM phenotypes with the highest number of gene associations. Any gene which is among the top 10 candidates for more than one disease is marked in bold. Catapult does make a great number of very reasonable predictions as observed below. For example, it seems quite likely that both insulin receptor (*INSR*, 3643) and insulin (*INS*, 3630) should be associated with insulin resistance, and that many growth factor receptors have been associated with various cancers.

**Table 2:** Top predictions for the Katz measure.

Leukemia	Alzheimer disease	Insulin resistance	Prostate cancer
MIM:601626	MIM:104300	MIM:125853	MIM:176807
*IL3* (3562)	*APLP2* (334)	*INS* (3630)	***BRCA1*** (672)
*SOCS1* (8651)	*HSPA8* (3312)	***AKT1*** (207)	*TP53* (7157)
***GRB2*** (2885)	*CTSB* (1508)	***INSR*** (3643)	*RAD51* (5888)
*NOP2* (4839)	*LRP1* (4035)	***GRB2*** (2885)	***EGFR*** (1956)
*CSF2RB* (1439)	*NID1* (4811)	***IGF1R*** (3480)	*ATM* (472)
*PPM1L* (151742)	*APOE* (348)	***CTNNB1*** (1499)	***AKT1*** (207)
*PTPN6* (5777)	*BDKRB2* (624)	*CREBBP* (1387)	*MAX* (4149)
*MYH11* (4629)	*PLAUR* (5329)	***PIK3CA*** (5290)	***CDK1*** (983)
*PPM1E* (22843)	*APLP1* (333)	*TYK2* (7297)	***PIK3CA*** (5290)
*PPM1B* (5495)	*CAV1* (857)	*GPD1* (2819)	*CSNK2A1* (1457)

Schizophrenia	Breast cancer	Gastric cancer	Colorectal cancer
MIM:181500	MIM:114480	MIM:137215	MIM:114500
*DRD2* (1813)	***BRCA1*** (672)	***GRB2*** (2885)	***PTEN*** (5728)
*AHCY* (191)	***IRS1*** (3667)	***EGFR*** (1956)	***CTNNB1*** (1499)
*ADRA2B* (151)	*MRE11A* (4361)	*NRAS* (4893)	***CDK1*** (983)
*XRN2* (22803)	***INSR*** (3643)	***IRS1*** (3667)	*GSK3B* (2932)
*MAT1A* (4143)	*CHEK1* (1111)	***MAPK1*** (5594)	*CDC20* (991)
*MAT2A* (4144)	*ATR* (545)	*PTPN11* (5781)	*PCNA* (5111)
*CHI3L2* (1117)	***PTEN*** (5728)	*HRAS* (3265)	*EGF* (1950)
*TSNAX* (7257)	***MAPK1*** (5594)	*MAP2K2* (5605)	*PTTG1* (9232)
*DDC* (1644)	*MAPK3* (5595)	*MAP2K1* (5604)	***IGF1R*** (3480)
*MAOB* (4129)	*UBE2I* (7329)	*SOS1* (6654)	*FOXO3* (2309)

Top 10 predictions not in the training set by Katz for the same eight OMIM phenotypes as in Table 1. Any gene which is among the top 10 candidates for more than one disease is marked in bold. The Katz method shows a weaker link between the number of diseases previously associated with a gene and its presence in the list, while still giving a number of very likely candidates.

The results for Catapult all seem very reasonable, from a biological standpoint. For example, Catapult identifies *APOE*, which even though is not linked to “Susceptibility to Alzheimer's disease” OMIM record (MIM:104300), is well known to be associated with Alzheimer's disease and is associated with two other OMIM records involving Alzheimer's (MIM:104310 and MIM:606889). BRCA1 is associated with “Breast-ovarian cancer, familial 1” (MIM:604370), not the record we show in Table 1 (“Breast cancer, susceptibility to”, MIM:114480), even so, it is ranked very highly among the candidate genes for breast cancer. Many of the other candidate genes listed are similarly very likely to be involved in the etiology of the diseases, like *TP53* and *KRAS* for many cancers. Indeed, what might be the most surprising about the results is how completely unsurprising they seem. Furthermore, there is a very high degree of overlap between the top predictions. Indeed, almost all the top 10 candidate genes for the eight diseases shown are shared between at least two of the eight diseases for Catapult. Moreover, when studying the results for the same diseases for ProDiGe, given in [15], we see the same pattern as we see for Catapult – a strong enrichment for genes that are already known to be associated with many diseases. For example, *EGFR* is predicted as a top ten candidate gene for gastric cancer by ProDiGe, as well as by Catapult (Table 1) and the Katz method (Table 2). In case of Alzheimer's disease, our methods and ProDiGe all predict *APOE* in the top ten.

However, the top predictions for both Catapult and ProDiGe seem to be governed more by what method is being tested than by what disease is being studied. For example, ProDiGe ranks *EXT1* in the top ten for six out of the eight diseases studied, and Catapult ranks *TP53* in the top ten for five of the diseases. In contrast, the results for the Katz measure (Table 2) exhibit a much lower degree of overlap between the top predictions. There is still a certain number of predictions shared, particularly between the different cancers and insulin resistance (type 2 diabetes). However, there is a good reason to believe that these shared genes actually reflect a common etiology, since epidemiological studies have shown a connection between the type 2 diabetes and cancers, in particular breast and colorectal cancer [30]. Overall, the predictions seem to reflect the relevance of a gene to the specific disease more than the overall likelihood that a gene is associated with *any* disease.

For example, many of the top ranked genes for Alzheimer's disease are related to amyloid precursor protein, *APP*, in various ways, such as *APLP2* and *APLP1*, which are homologs of *APP*, *CTSB*, also known as amyloid precursor protein secretase, *LRP1*, which is necessary for clearance of *APP* plaques, and *APOE*, apolipoprotein E. A recent review of the role *APP* and its interaction partners play in Alzheimer's disease can be found in [31]. *CAV1* has also recently been studied in relation to *APP* and Alzheimer's disease [32]. Another interesting candidate Alzheimer's disease is *BDKRB2*, bradykinin receptor B2. *BDKRB2* is highly expressed in the central nervous system according to the Human Protein Atlas, and modulation of *BKRB2* results in a cellular state highly enriched for amyloid 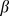 peptide in a skin fibroblast cell line from a patient with early onset familial Alzheimer's disease [33].

We see a similar pattern with more specific predictions that still seem well supported by the literature for most of the other diseases in Table 2. For example, the association of *MYH11* with leukemia, through inversion of a region on chromosome 16 and the formation of a *CBFB*–*MYH11* chimera, is well known and was first identified in [34]. However, it is not associated with the OMIM record shown in Table 2. For schizophrenia, the top ranked candidate gene, *DRD2*, is well known to be associated with schizophrenia (under MIM:126450), and a recent study has highlighted a potential role for *ADRA2B* in schizophrenia [35].

### Validation on singletons highlights methods that detect novelty

The cross-validation evaluation shown in Figures 3 and 4 clearly shows that Catapult is better at recapitulating the genes known to be involved in a disease than any of the other methods. However, recapitulation of previously known results is rarely the goal in biology. We therefore seek a measure that would reflect how suited a method is for correctly identifying associations between diseases and genes that are less well studied.

There are two ways one could envision for doing this in a cross-validation framework – either one could hide *all* associations between a given gene and diseases, thereby hoping to put it on equal footing with genes still unstudied, or one restricts the cross-validation to genes that are only associated with a single phenotype. There are clear advantages to both approaches. The former approach allows us to do validation on a larger set, namely all known gene-disease associations, and thereby reach stronger statistical strength. The latter approach has more subtle, but in our opinion greater, advantage. The biases that favor already well studied genes are not only present in the gene-disease association data, but also in the data that we use for constructing the networks and the model species data sets. This gives rise to small differences between genes that have been the well studied genes and the poorly characterized genes. By only looking at the least studied genes in our data set for which we do have known gene-disease associations, we can minimize the risk that any signal that we detect is merely some general characteristic of well studied genes, and instead evaluate how well a method can detect truly novel gene–disease associations.

We tested all the methods using cross-validation restricted to genes with only a single disease (or drug) association (which we call *singletons*, shown in yellow in Figure 9). The results of this cross-validation are presented in Figure 6. Catapult still does much better than ProDiGe (the only other supervised method) but does worse than the unsupervised methods, in contrast to Figure 4 (that uses the same setting for all the methods). The PRINCE and ProDiGe methods are consistent with (and sometimes perform slightly better than) the three-fold cross-validation evaluation. RWRH and the Katz measure perform better than the supervised learning methods ProDiGe and Catapult in this evaluation sheme. The fact that PRINCE performs so well on singletons when evaluated on drug data is surprising, given that the only information it uses is the HumanNet gene network. Simpler random-walk based methods in general perform better than the supervised counterparts, and do so consistently in two completely distinct data sets. Furthermore, we find that the qualitative results of the methods (Tables 1 and 2) indicate that the supervised Catapult tends to emphasize the same “common” genes much more than the Katz measure does, which is consistent with the difference in performance we observe between the cross-validation on the full set of gene-disease associations and cross-validation restricted to the singleton genes.

The differences in performance between the full test set and the singletons raise the question of what we really are trying to do when we predict gene-phenotype associations. Ultimately, the correct evaluation criterion for gene-phenotype association predictions must be successful laboratory confirmation of the predictions. However, lacking that, we often resort to different cross-validation schemes to measure how well a method does. We have here shown that even quite modest changes in the evaluation scheme can alter the relative performance of the methods tested. In the case of Catapult and the Katz measure, this is likely because the tendency of “common” genes to be involved in diseases in general is a property that the supervised Catapult can easily detect and make use of, which strongly boosts its performance on the full data set but does nothing for its performance on the singleton genes. By actively restricting the use of features that are characteristic of “common” genes (For instance by the restricting the maximum allowed path length), we can counteract this tendency at the cost of performance in the full cross-validation (data not shown).

### Conclusions

We have proposed two methods for inferring gene-phenotype associations, Katz and Catapult. Katz is motivated by social network link prediction and Catapult is a supervised extension to Katz which learns the weights for walks that have different lengths and that involve different kinds of data. While Catapult significantly outperforms other state-of-the-art gene-phenotype association methods using a conventional cross-validation evaluation strategy, such a cross-validation strategy does not necessarily reflect the properties of a scenario in which one wants to predict *novel* gene-phenotype associations involving less studied genes.

To address such cases, we propose a cross-validation approach restricted to relatively little studied genes. In this framework the Katz method and the related RWRH and PRINCE methods do better than the supervised methods, indicating that if the objective is to find new gene-disease or gene-drug associations involving genes not yet well studied, these approaches are more appropriate.

We therefore conclude that comparisons of gene-phenotype methods do not necessarily lead to a simple ordering from best to worst. If the goal of a researcher is to find new directions for research or find previously unknown biology, she might not want to use methods that perform the best in a conventional cross-validation framework. For instance, she might prefer a method like the Katz measure, which does better when tested on genes only associated with a single disease, to a method like Catapult, which emphasizes genes that are important in general. In the future, it is therefore important that descriptions of new gene-phenotype association methods include a careful discussion on how the method is intended to be used.

## Materials and Methods

### Gene Networks

We use two sources of gene-gene interactions in our experiments.

1. **HumanNet** [**14**] **:** A large-scale functional gene network which incorporates multiple data sets, including mRNA expression, protein-protein interactions, protein complex data, and comparative genomics (but not disease or phenotype data). HumanNet contains 21 different data sources, which are combined into one integrated network using a regularized regression scheme trained on GO pathways. The edges in the network have non-negative edge weights, and there are 733,836 edges with non-zero weights.
2. **HPRD network** [**36**] **:** Most of the published work on predicting gene-disease associations [5]–[8], [37] use the HPRD network. The network data was downloaded from [4]. The edges in the HPRD network are unweighted, and the network is much sparser than HumanNet. In particular, the HPRD network has 56,661 associations compared to 733,836 (weighted) associations for HumanNet.

### Phenotypes from other (non-human) species

We collected gene-phenotype associations from literature and public databases for eight different (non-human) species: plant (*Arabidopsis thaliana*, from TAIR [38]), worm (*Caenorhabditis elegans* from WormBase [39], [40]), fruit fly (*Drosophila melanogaster* from FlyBase [41]), mouse (*Mus musculus* from MGD [42]), yeast (*Saccharomyces cerevisiae* [10], [43]–[45]), *Escherichia coli* [46], zebrafish (*Danio rerio* from ZFIN [47]), and chicken (*Gallus gallus* from GEISHA [48]). We determined orthology relationships between genes in model species and human using INPARANOID [49]. Detailed description on the extraction of most datasets can be found in [16] and the resulting dataset has been summarized in Table 3.

**Table 3:** Species used.

Index	Species	# Phenotypes	# Associations
1	Human (*Hs*)	3,209	3,954
2	Plant (*At*)	1,137	12,010
3	Worm (*Ce*)	744	30,519
4	Fly (*Dm*)	2,503	68,525
5	Zebrafish (*Dr*)	1,143	4,500
6	*E.coli* (*Ec*)	324	72,846
7	Chicken (*Gg*)	1,188	22,150
8	Mouse (*Mm*)	4,662	75,199
9	Yeast (*Sc*)	1,243	73,284

Different species used for inferring gene-phenotype associations in the proposed methods Katz and Catapult, and sizes of the gene-phenotype networks for the species, restricted to orthologs of human genes. The total number of human genes with any kind of phenotype annotation is 12331.

*E. coli* phenotypes were obtained from the file ‘coli_FinalData2.txt’ on May 20, 2011 [46]; we sorted each gene's phenomic profile by score, taking both the top and bottom forty conditions and assigning them to the gene. Thus, we considered each condition to be a phenotype, and the genes associated with that phenotype were those genes whose growth was most affected (either positively or negatively) in the corresponding condition. As a proxy for chicken phenotypes, tissue specific mRNA expression patterns were derived from GEISHA *in situ* hybridization annotations, which were kindly provided in XML format on June 24, 2011. Genes were sorted into multiple bins by stage, by location, and by location and stage together. If there were more than fifty genes in a specific location and more than three at a specific stage at that location, a new phenotype was created (“*anatomical location* at stage 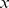
”); regardless, each location became a phenotype. Worm phenotypes [40] were divided into two datasets, ‘green-broad’ and ‘green-specific’, based on the broad and specific phenotypes. GO biological processes from TAIR and ZFIN were processed in the same manner. We kept only those annotations with evidence codes IMP, IDA, IPI, IGI, TAS, NAS, IC, and IEP. For TAIR, we used ‘ATH_GO_GOSLIM.txt’, downloaded on August 23rd, 2010; and for ZFIN, we obtained GO biological processes from geneontology.org (‘gene_association.zfin.gz’) on April 26th, 2011.

### Evaluation data

We perform experiments on two types of data sources:

- **OMIM Phenotypes:** We obtained new OMIM data from the Online Mendelian Inheritance in Man (OMIM) project [27] on August 11, 2011. OMIM phenotypes have become the standard data set for the evaluation of prediction of gene-disease associations[5]–[8], [15], [37]. All the compared methods use similarities between phenotypes [28] to form the (weighted) phenotype-phenotype network 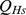.
- **Drug data:** This includes four benchmark data sets of Drug-Target interactions in humans involving enzymes, ion channels, G-protein-coupled receptors (GPCRs) and nuclear receptors, first studied in [50]. Refer to Table 4 for statistics on the data sets. The data sets were made available by [19] and downloaded from [51].

**Table 4:** Benchmark Drug data sets used for evaluation.

Index	Type	# Drugs	# Associations
1	Enzymes	445	2,926
2	Ion Channels	210	1,476
3	GPCRs	223	635
4	Nuclear Receptors	54	90

### Problem setup and Notation

Let 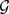 denote the set of human genes and for each species 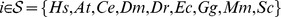, let 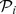 denote the set of phenotypes for the species. Refer Table 3 for descriptions of the species and a summary of the data sets. Also, let 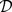 denote the set of drugs (*i.e.* the four benchmark data sets mentioned in Table 4). For each species 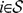, we constructed a gene-phenotype association matrix 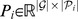, such that 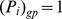 if gene 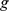 is associated with phenotype 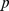 or 0 otherwise. For methods using multiple species, we used 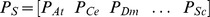 and recall that 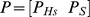 in equation (5). Similarly, we constructed a drug-gene interaction matrix 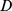 using drugs data where 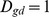 if gene 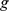 is known to be associated with drug 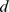 (note that 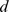 can be one of enzymes, ion channels, GPCRs or nuclear receptors) and 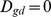 otherwise. Using the two types of gene-gene interaction data HPRD and HumanNet, we constructed matrices 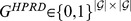, and 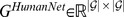 respectively. We constructed a phenotype-phenotype network 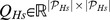 (*i.e.* corresponding to humans) using OMIM phenotype similarities [28]. For experiments with drug data, we did not have access to any such similarity score for drug pairs, so we set the drug-drug network to 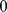. The same is the case for other species data as well, and we set the corresponding entries in 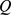 to be 0, both for the experiments with OMIM and for the drug data. Following the approach by Vanunu et al. [7], we apply a logistic transformation to the similarities 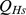, *i.e.* 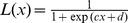 where 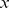 represents an entry of 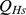. For setting 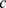 and 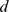, see [7]. We use the transformed 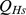 network in all our experiments.

#### The CATAPULT algorithm

Catapult uses a biased SVM framework to classify gene-phenotype pairs of humans with a single training phase, thereby making use of the relation between different phenotypes. Recent work [25] uses the *bagging* technique to obtain an aggregate classifier using positive and unlabeled examples. In this approach, one draws a random bootstrap sample of a few unlabeled examples from the set of all unlabeled examples and trains a classifier treating the bootstrap sample as negatives along with the positive examples. Bagging helps to reduce the variance in the classifier that is induced due to the randomness in the “negative” samples. Let 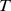 be the number of bootstraps, let 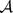 be the set of positives (*i.e.* gene-phenotype pairs that correspond to known associations), let 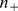 denote the number of examples in 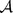, and let 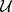 denote the set of unlabeled gene-phenotype pairs. We train a *biased* SVM, where we use a penalty 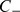 for false positives and relatively larger penalty 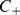 for false negatives.

The bagging algorithm that trains and combines several biased SVM classifiers used by Catapult is as follows:**initialize** 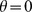, 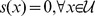, and 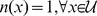.**for** 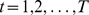:

1. Draw a bootstrap sample 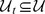 of size 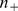.
2. Train a linear classifier 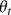 using the positive training examples 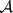 and 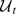 as negative examples by solving:

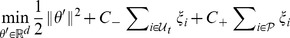
(6)

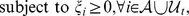


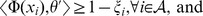


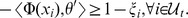

1. For any 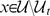 update:

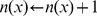
.

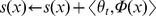
.

**return** 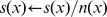, 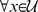.

We train a biased SVM given in equation (6) during each iteration using all the known positive examples in 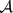 and a randomly chosen set of “negatives” 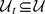. Positive and negative examples may not be linearly separable, and the usual approach in SVMs is to penalize each example based on how far it is from meeting its margin requirement, through the use of *slack* variables 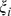. The scoring function for iteration 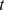 is proportional to the distance of the point 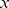 from the hyperplane and is given by the standard dot product,
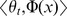
where 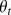 is the normal to the hyperplane learned using the random bootstrap at the 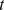
th iteration and 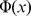 is the feature vector corresponding to 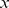. For small number of boostraps, say 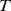 in the range 10-100, 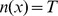 for most of the unlabeled examples and thus the procedure in effect scores (most of the) unlabeled examples using the average hyperplane 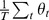. We set 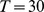 in our experiments. Recall that, in our framework, an instance 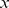 corresponds to a gene-phenotype *pair*. In contrast to the traditional SVM classifiers that classify a pair as positive or negative based on the sign of 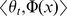, we use the value as a score under the assumption that the further a point is on the positive side of the hyperplane, the more likely it is to be a true positive.

#### Parameters

In equation (6), 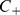 and 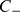 are the penalties on misclassified positives and negatives respectively. Typically, 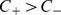. The weights control the relative widths of the margins on either sides of the hyperplane. As 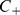 increases from 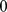 to 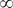, the margin on the side of the positive examples shrinks, and as 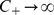, the classifier attempts to make *no* mistake on the positive examples. The ratio 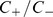 determines the “weight” of a positive example, and we want this to be high. In our experiments, we set 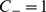 and 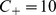, which is found to be the best by cross-validation. The cross-validation procedure for tuning parameters is given as follows:

1. Sample a fraction (70%) of the positives from the training data points (gene-disease associations) to form the validation set 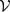.
2. Split the validation set into 5 folds: 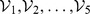.

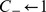
.
4. **for** 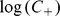 **in** 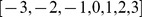:

**for** 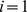 **to** 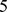:

- Fix 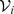 to be the test set and the remaining 4 folds, 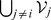, to be the training set.
- Train Catapult with the positive training set 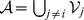, and the current 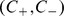 values in equation (6). (Note that an equal number of “negative” examples are randomly sampled from the unlabeled gene-disease associations.).
- Obtain the recall rate 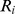 of Catapult on the hidden test set, *i.e.* fraction of true positives in 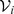 identified in the top 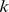 predictions, where 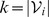.

Let 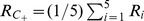.

1. **Return** 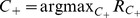 and 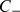.

Using the cross-validated values of 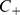 and 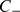 for a particular train-test split (Fixing 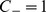, 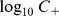 was varied in the range 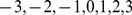 and 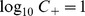 was typically the best), we train the model on the training data and evaluate on the test data. This is done three times corresponding to 3 random train-test splits of the full gene-disease associations data.

#### Features derived from hybrid walks

Before applying any supervised machine learning approach, we need to construct *features* for gene-phenotype pairs. The features that we use are all based on paths in the heterogeneous network. Recall that in the Katz measure, the weights for combining the contributions from walks of different lengths are fixed beforehand. We observe from equation (5) that, for a given length of walk, there are multiple ways of obtaining hybrid walks, as given by the terms in the series. For a given gene-phenotype pair, different walks of the same length, and walks of different lengths can be used as features for the pair. Thus learning a biased SVM provides an efficient way to learn the weights, and could help improve on the prediction performance over a particular choice of weights, say, 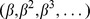 as in the Katz measure. Clearly, the dimensionality of the feature space is exponential in 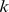, the length of the walk, and makes us vulnerable to the curse of dimensionality because the examples are limited. However, taking a cue from the fact that the weights of increasing walk lengths need to be heavily damped, we ignore higher order terms and thereby keep the dimensionality of the feature space small.

#### Relationship between the learned 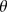 and 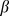


The complete set of features used by Catapult and the corresponding weights learned are listed in Table 5. The features relate to the expression for the Katz method given in (5). In particular, the term involving 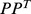 can be written as 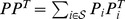, where 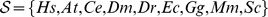. The Katz method weighs terms involving paths of length 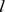 by 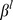, and does *not* distinguish between species or between sources. As an example of distinguishing by *source*, consider the terms corresponding to 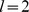 in the expression (5), i.e. 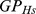 and 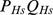. The Katz method weighs both the types of paths by 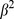 whereas the Catapult method learns two different weights 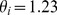 and 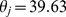 corresponding to the two features, as observed from Table 5. For distinguishing by species, consider the term 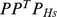 corresponding to 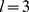 in (5). In this case, Katz method uses 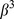, whereas Catapult learns a set of 9 different weights corresponding to different species, i.e. 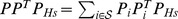. We observe from Table 5 that different species contribute differently towards the final prediction. Furthermore, we also observe from our experiments using species-wise features not only lends interpretability but also improves the accuracy of the predictions, as compared to combining features corresponding to same walk lengths (Note that all Catapult results shown in the paper use the features listed in Table 5, and results for combining features are not shown).

**Table 5:** Weights by Catapult.

Type	Feature	Learned weights	Feature	Learned weights
	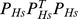	31.04	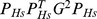	8.97
Human	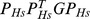	2.60	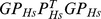	5.98
	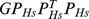	1.00	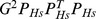	3.31
	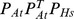	8.09	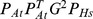	0.74
Plant	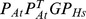	1.06	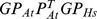	2.18
	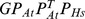	1.20	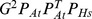	0.65
	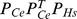	5.75	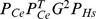	0.33
Worm	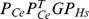	0.69	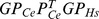	0.55
	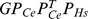	0.62	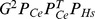	0.29
	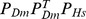	4.58	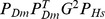	0.90
Fly	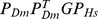	0.93	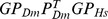	1.36
	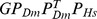	0.72	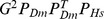	0.55
	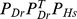	8.28	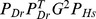	1.16
Zebrafish	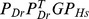	0.77	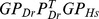	2.68
	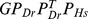	0.52	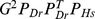	0.69
	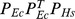	1.67	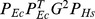	0.19
*E. coli*	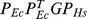	0.30	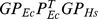	0.75
	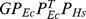	0.29	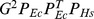	0.12
	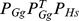	3.76	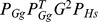	0.32
Chicken	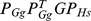	0.30	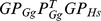	1.35
	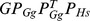	0.23	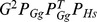	1.82
	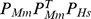	15.03	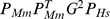	1.54
Mouse	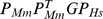	1.35	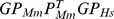	2.13
	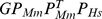	0.83	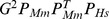	0.70
	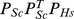	5.55	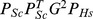	0.30
Yeast	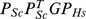	0.61	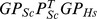	0.59
	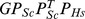	0.56	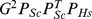	0.25
Gene network	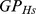	1.23	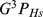	0.57
	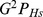	3.52	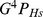	0.36
Phenotype network	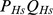	39.63	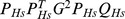	4.28
	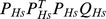	21.02	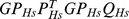	2.56
	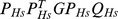	1.70	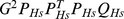	1.43
	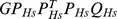	0.64		

Weights learned for different features by Catapult using the biased SVM with bagging procedure, using the HumanNet gene network. Two important observations are: (1) Features corresponding to longer path lengths receive relatively much smaller weights (note that path length can be deduced from the number of terms in the feature, for example, 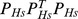 has path length 3, while 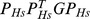 has path length 4). (2) Features corresponding to different species receive different weights, in particular, features derived from mouse phenotypes get the highest weights, which makes sense given the relative evolutionary proximity between humans and mice. The relative weights of different information sources are not straightforward to interpret. However, we can see that some higher order features are informative.

#### Random Walks with Restart on Heterogeneous network

Random Walks with Restart on Heterogeneous network (RWRH) is an algorithm for predicting gene-disease associations proposed by Li and Patra [8]. RWRH performs a random walk on a heterogeneous network of gene interactions (HPRD) and human diseases (we used OMIM phenotypes and the drug data described above). The method constructs a heterogeneous network using 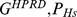 and 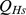 networks and runs a personalized PageRank computation, a popular choice for ranking documents and web pages, on the network. The random walk is started from a set of seed nodes, which for a phenotype 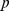 is the set of genes known to be associated with 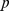, and gene nodes are ranked by the probability that a random walker is at a given gene, under the steady state distribution for the random walk. In particular, RWRH [8] considers the following heterogeneous network:
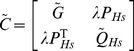
(7)where 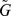 is the gene-gene interactions matrix, 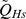 is the phenotype-phenotype similarity matrix, and 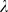 is the probability that the random walker jumps from a gene node to a phenotype node (or vice versa). The matrices 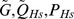 and 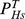 are normalized by row-degree. The rows of the matrices 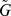 and 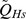 scaled by a factor of 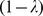, as appropriate, so that 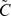 is a row-stochastic matrix, *i.e.* 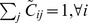. In [8], 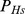 is the gene-disease association matrix corresponding to OMIM phenotypes, 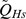 is the corresponding similarity matrix, and 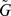 is derived from 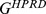. Genes are ranked for a given disease 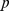 using the steady state vector 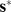, the solution to the equation:
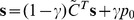
(8)where 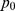 is the restart vector (indicator vector of the set of seed nodes known to be associated with 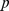) and 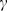 is the restart probability. In our experiments, we use OMIM phenotypes matrix 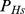 as well as the gene-drug interaction matrix 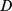, and two types of gene-gene matrices to derive 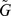. Recall that in the latter case, we do not have similarity information for drugs, and therefore we set drug-drug similarity matrix to 0. It is also straightforward to incorporate phenotype data from multiple species in the method, by replacing 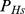 with 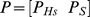, analogous to our Katz method.

#### PRINCE

The PRINCE method, proposed by Vanunu et al. [7], is another graph-based method that can be thought of as a special case of RWRH. Here, the random walk is only over the gene interaction network instead of the heterogeneous network. The phenotype similarities are incorporated in the restart vector. The vector of scores computed by PRINCE for a given phenotype 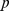 can be expressed as
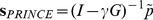
(9)where 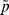 is the smoothed phenotype, *i.e.* 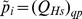 where 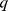 is the phenotype most similar to 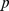 such that gene 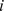 is known to be associated with disease 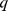 and 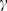 is the restart probability. Note that, similarly, the scores computed by RWRH can be written succinctly as
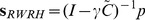
(10)where 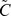 is defined in equation (7). We show the relationship between the Katz method and other random-walk based methods (PRINCE and RWRH) in the Text S1. The absence of similarity information for other (non-human) species phenotypes and drugs renders direct extension of PRINCE to multiple species data inconsequential. We must emphasize here that PRINCE does not allow walks through the gene-phenotype interaction network or the phenotype-phenotype interaction network. As a result, availability of other species data becomes irrelevant when predicting genes for a given disease (or other drug data in case of predicting for a given drug).

#### ProDiGe

The ProDiGe method, proposed by Mordelet and Vert [15], makes use of positive-unlabeled learning and a multiple kernel learning framework to integrate information from multiple types of gene interaction data and phenotype similarities. Kernels are defined over pairs of genes and pairs of phenotypes, and the kernel value for a pair of gene-phenotype pairs is derived using the individual gene and phenotype kernels. In particular, let 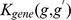 denote the kernel for genes, and 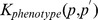 denote that for phenotypes. Then, the kernel for the pairs 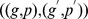 is simply,
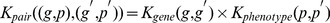
(11)


The gene-phenotype pairs are then classified using a support vector machine using the constructed kernel. Note that ProDiGe [15] does not use any other species phenotype information, but only the OMIM phenotypes. In our experiments on OMIM phenotypes, we used the kernels 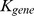 and 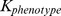 provided by Mordelet [15] downloaded from:

http://cbio.ensmp.fr/jvert/svn/prodige/html/prodige-0.3.tar.gz. For the drug data, we used a simple Dirac kernel (since kernel matrices need to be positive definite) for 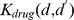, *i.e.*

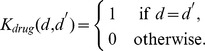



### Implementation

We implemented all the methods in Matlab. Our implementation of Catapult can be downloaded from the Catapult home page: http://marcottelab.org/index.php/Catapult. A web interface for querying recommendations for a given phenotype is also accessible from the page. Obtaining features for all gene-phenotype pairs takes about 20 minutes. Training Catapult is much faster, and takes a few seconds per iteration of the algorithm on our cluster machines (2.8 GHz processor, 32GB RAM). The sourcecodes for Li and Patra's RWRH method and ProDiGe were obtained from the respective project home pages http://www3.ntu.edu.sg/home/aspatra/research/Yongjin_BI2010.zip and http://cbio.ensmp.fr/jvert/svn/prodige/html/prodige-0.3.tar.gz For PRINCE, we use MATLAB code kindly provided by Oded Magger.

## Supporting Information

Text S1 **A detailed derivation of the relationship between Katz on the heterogeneous network, PRINCE, and RWRH.**
(PDF)
