# Correction: Prediction and Validation of Gene-Disease Associations Using Methods Inspired by Social Network Analyses

**DOI:** 10.1371/annotation/5aeb88a0-1630-4a07-bb49-32cb5d617af1

**Published:** 2013-09-12

**Authors:** U. Martin Singh-Blom, Nagarajan Natarajan, Ambuj Tewari, John O. Woods, Inderjit S. Dhillon, Edward M. Marcotte

The following was incorrectly appended to the Abstract, and should be read as the Acknowledgement Statement as is already indicated: "The authors want to thank Jon Laurent and Kris McGary for some of the data used, and Li and Patra for making their code available. Most of Ambuj Tewari's contribution to this work happened while he was a postdoctoral fellow at the University of Texas at Austin." 

