# Supplementary material for: Prediction and Validation of Gene-Disease Associations Using Methods Inspired by Social Network Analyses
Source: PLoS One. 2013 May 1;8(5):e58977. doi: 10.1371/journal.pone.0058977 (PMC3641094; doi:10.1371/journal.pone.0058977)
Supplement: Text S1 — A detailed derivation of the relationship between Katz on the heterogeneous network, PRINCE, and RWRH. (PDF) [file pone.0058977.s001.pdf]

Supplementary Material

Relationship between Katz on the heterogenous network and RWRH

Restricting P to human phenotypes, *i.e.* letting $P = P_{Hs}$, weighing P and $P^\top$ by λ in the heterogeneous network C where $0 < \lambda < 1$ is the jump probability, in Equation (3), and appropriately normalizing the matrices by row-degrees and scaling, we get the heterogeneous network construction $\widetilde{C}$, in Equation (7), used in the RWRH method [8]. The RWRH method, when extended to our heterogenous network, turns out to be *equivalent* to the Katz measure provided the columns of the combined matrix C are normalized appropriately. The equivalence is shown below. Let C^N denote the *normalized* matrix, with the different blocks weighted as described above. Then, the column corresponding to a gene g in the matrix C^N (*i.e.*, one of the first $|\mathcal{G}|$ columns), written $C^N_{:,g}$, is given by:

$$C^N_{:,g} = \begin{bmatrix} \lambda \frac{G_{:,g}}{\|G_{:,g}\|_1} \\ (1-\lambda)\frac{P^\top_{g,:}}{\|P_{g,:}\|_1} \end{bmatrix},$$

and the column corresponding to a phenotype p in the matrix C^N(*i.e.*, one of the remaining $|\mathcal{P}_{Hs}|$ columns), written $C^N_{:,p}$, is given by:

$$C^N_{:,p} = \begin{bmatrix} (1-\lambda)\frac{P^\top_{:,p}}{\|P_{:,p}\|_1} \\ \lambda \frac{Q^\top_{:,p}}{\|Q_{:,p}\|_1} \end{bmatrix}.$$

Note that if a gene g is not known to be associated to any phenotype (*i.e.* $\|P_{g,:}\| = 0$) then we will simply use $\lambda = 1$ for g. Case $\|Q_{:,p}\| = 0$ is handled similarly. Then we consider the evolution:

$$\mathbf{s}_{T+1} = \beta C^N \mathbf{s}_T + (1-\beta)C^N_{:,p}$$

where $C^N_{:,p}$ is simply a probability distribution with equal mass on all genes known to be associated with a phenotype p of interest, and mass on the diseases related to p . The genes are then ranked in the order of the mass that is assigned to them under the steady state distribution $\mathbf{s}$ of this evolution. The steady state vector $\mathbf{s}$ should satisfy

$$\mathbf{s} = \beta C^N \mathbf{s} + (1-\beta)C^N_{:,p}$$

which readily yields

$$\mathbf{s} = (1-\beta)[I - \beta C^N]^{-1} C^N_{:,p} \ .$$

Thus the score matrix computed by RWRH can be written as[3],

$$\beta[I - \beta C^N]^{-1} C^N = \beta C^N + \beta^2 (C^N)^2 + \beta^3 (C^N)^3 + \dots \ .$$

which is *exactly* Katz but on the *normalized* matrix C^N instead of C itself.

Relationship between Katz on the heterogenous network and PRINCE

Examining the computation of Katz on heterogeneous network closely yields an interesting connection to PRINCE. As $k \to \infty$ in Equation (4) and for appropriate choice of β, let

$$S^{katz}(C) = (I - \beta C)^{-1} = \begin{bmatrix} S_{GG} & S_{GP} \\ (S_{GP})^\top & S_{PP} \end{bmatrix} \ ,$$

[3]Multiplying either sides of the equation by constant factor $\beta/(1-\beta)$ does not affect the ranking of candidates.

where it can be shown that

$$S_{GP} = S^{katz}(G)P\left[I - (Q + P^{\top}S^{katz}(G)P)\right]^{-1} \ . \tag{12}$$

Note how the Katz similarity matrix $S^{katz}(G) = (I - \beta G)^{-1}$ for the gene-gene network G itself appears in the expression above. The expression above takes into account all kinds of paths in the combined network that start in gene nodes and end up in human phenotype nodes. The corresponding score matrix computed by PRINCE [7] method can be generalized as

$$S_{GP}^{PRINCE} = S^{Katz}(G)PQ \ . \tag{13}$$

Note that it is a form of generalization — PRINCE "smoothes" a given phenotype using its most similar neighbor, whereas the term PQ in Equation (13) combines all the neighbors linearly. Also note that the expression should strictly have P_{Hs} and Q_{Hs} instead of P and Q as PRINCE [7] uses only human phenotypes data. However, using P and Q in Equation (13) enables comparison to the expression corresponding to the Katz method given in Equation (12). Clearly, Katz on the heterogeneous network C generalizes PRINCE method. In particular we observe that while PRINCE relies on the matrix Q to obtain "smoothed" phenotypes by sharing information across phenotypes, Katz on the heterogeneous network uses a combination of Q and $P^{\top}S^{katz}(G)P$.
